# Bariatric Surgery Has a Long-Term Beneficial Impact on Urinary Incontinence in Women with Obesity

**DOI:** 10.3390/medicina61040564

**Published:** 2025-03-22

**Authors:** Magdalena Sternau, Mateusz Czajkowski, Piotr Wierzbicki, Marzena Kogut-Wierzbicka, Karolina Zarzyka, Maciej Milewczyk, Krzysztof Czurak, Monika Proczko-Stepaniak, Marcin Matuszewski

**Affiliations:** 1 Department of Urology, Medical University of Gdańsk, 80-210 Gdańsk, Poland; mateusz.czajkowski@gumed.edu.pl (M.C.); krzysztof.czurak@gumed.edu.pl (K.C.); marcin.matuszewski@gumed.edu.pl (M.M.); 2 Department of Histology, Medical University of Gdańsk, 80-210 Gdańsk, Poland; pwierzb@gumed.edu.pl; 3 Faculty of Medicine, Academy of Applied Medical and Sciences, 82-300 Elbląg, Poland; m.kogut-wierzbicka@amisns.edu.pl; 4 Department of Urology, F. Ceynowa Specialist Hospital, 84-200 Wejherowo, Poland; lozycakarolina@gmail.com; 5 Department of Urology and Oncological Urology, Specialist Hospital in Kościerzyna, 83-400 Kościerzyna, Poland; maciej.milewczyk@gumed.edu.pl; 6 Department of General, Endocrine and Transplant Surgery, Medical University of Gdańsk, 80-210 Gdańsk, Poland; mproczko@gumed.edu.pl

**Keywords:** urinary incontinence, bariatric surgery, obesity, woman with obesity, sleeve gastrectomy, gastric bypass

## Abstract

*Background and Objectives*: To evaluate the long-term efficacy of bariatric surgery in ameliorating urinary incontinence in women with obesity. Additionally, to assess the impact of comorbidities on the persistence of symptoms and compare the effectiveness of two types of bariatric interventions. *Materials and Methods*: This prospective, single-centre study included 124 women with preoperative urinary incontinence (UI). A total of 92 (74.19%) responded to follow-up and underwent laparoscopic sleeve gastrectomy (LSG) (*n* = 52; 56.52%) or one anastomosis gastric bypass (OAGB) (*n* = 40; 43.48%). The cohort was divided into stress urinary incontinence (SUI) (*n* = 57; 61.96%), mixed urinary incontinence (MUI) (*n* = 33; 35.87%), and urge urinary incontinence (UUI) (*n* = 2; 2.17%). Before surgery, patients were assessed for comorbidities and completed the International Consultation on Incontinence Questionnaire-Urinary Incontinence Short Form (ICIQ-UI SF) (score range 0–21) and the Urogenital Distress Inventory (UDI-6) (score range 0–100) questionnaires. After 5 years, the patients completed the same questionnaires again for the final assessment. *Results*: Bariatric surgery demonstrated a statistically significant reduction in UI symptoms (*p* < 0.001), with a more pronounced improvement in SUI than in MUI, and with complete resolution in patients experiencing UUI. LSG was more effective than OAGB at alleviating UI (*p* < 0.001 vs. *p* = 0.017). Notably, childbirth, particularly vaginal delivery, was associated with a higher risk of persistent UI after surgery (*p* = 0.025). The correlation between postoperative BMI and improvement in UI symptoms was not statistically significant (*p* = 0.64). *Conclusions*: Bariatric surgery provides a beneficial secondary effect on urinary incontinence (UI) in women with obesity who undergo the procedure for obesity. The LSG method is superior to OAGB when considering the improvement in incontinence symptoms. Furthermore, the LSG procedure should be considered the primary choice for women with obesity experiencing UI with a history of vaginal delivery.

## 1. Introduction

Obesity is a growing global concern. According to estimates, the prevalence of obesity in Europe is expected to increase by 2025. In particular, individuals aged 10–19 years have experienced a notable increase in obesity rates. The Body Mass Index (BMI) used to determine obesity is sex- and age-independent, and is defined as a total BMI of equal or greater than 30 kg/m^2^. According to this definition, it is estimated that approximately 23% of adults in Europe are obese. The prevalence of obesity is higher in women (24%) than in men (22%). The World Health Organization (WHO) identified two primary causes of obesity. The first factor is associated with exposure to obesity before and during pregnancy. The second involves an unhealthy diet, insufficient physical activity, and exposure to obesogenic environmental factors. Obesity is a significant risk factor for various diseases such as type 2 diabetes mellitus, cardiovascular diseases, and musculoskeletal complications. Furthermore, it can contribute to the development of 13 different types of cancer, including breast, colorectal, renal, and uterine cancers [1].

Urinary incontinence (UI) is a health issue that primarily affects women, with a prevalence rate of approximately 16.1 to 68.8%, and tends to worsen with age. Stress urinary incontinence (SUI) is the most common form of this condition, followed by mixed urinary incontinence (MUI), and urge urinary incontinence (UUI). The most significant risk factors for UI in women are age, obesity, vaginal delivery, menopause, chronic obstructive pulmonary disease, diabetes mellitus, hypertension, and smoking [2]. Obesity is a significant modifiable risk factor for urinary incontinence, as it leads to increased intraabdominal and intravesical pressure, which puts additional stress on the pelvic floor. Although the precise mechanism remains unclear, it is believed to cause increased urethral mobility and detrusor instability. Additionally, obesity can result in muscle weakness and ligament instability owing to prolonged strain and stretching. Furthermore, after weight loss there are observed changes in urodynamic measures, such as decreased initial and maximum capacity intravesical pressure and increased Valsalva leak point pressure [3,4]. Both urinary incontinence and obesity can have a negative impact on mental health and quality of life, as they are associated with higher risks of anxiety, depression, and sleep disorders [3,5]. Urinary incontinence can be treated with a range of therapeutic alternatives that are dependent on the nature of the condition. Nevertheless, weight loss may serve as an effective intervention for all UI subtypes, given that obesity is a modifiable risk factor for UI [6].

Obesity can be managed using various methods, including bariatric surgery. Indications for bariatric surgery are established by analyzing the BMI, age, and comorbidities of patients. It is currently recommended for patients with BMI ≥ 30 kg/m^2^. According to the American Society for Metabolic and Bariatric Surgery (ASMBS), even in Class I obesity (BMI 30–34.9 kg/m^2^), metabolic and bariatric surgery (MBS) should be considered for patients who do not achieve anticipated or sustained weight loss or improvement of comorbidities, particularly diabetes [7]. Bariatric treatment has the potential to result in substantial weight loss within a six-month period, which can also lead to a decrease in the incidence of comorbidities and all-cause mortality, and an improvement in overall quality of life [8].

There has been insufficient research on the impact of bariatric surgery on urinary incontinence, particularly with regard to large participant groups and extended follow-up periods [9,10]. Notably, no prior studies have investigated outcomes according to specific types of bariatric surgery. Recent studies have analyzed individuals from various regions, including Asia [11,12], North America [13], South America [14], and Western Europe [9]. Given the differences in medical care practices between individual European countries and other parts of the world, our objective was to evaluate the population of Central Europe, which had not yet been examined.

Despite this knowledge gap, most studies have demonstrated that bariatric procedures result in a significant reduction in urinary incontinence [10,11,14]. However, it remains uncertain whether this effect is short-term or not. In addition, some studies do not support this claim [15].

Therefore, due to discordance among the aforementioned research results, we initiated a prospective study with a lengthy follow-up period of five years, encompassing a substantial cohort of patients, and assessed the two types of bariatric procedures. Consequently, our research hypothesis aimed to assess the long-term efficacy of bariatric surgery in reducing urinary incontinence symptoms over a 5-year period. Furthermore, we sought to determine the potential influence of comorbidities (diabetes mellitus type II, hypertension, tachycardia, hypercholesterolemia, sleep apnea, and menopause), modes of delivery (vaginal and C-sections), and other abdominal or gynecological operations on the outcomes. Lastly, we aimed to investigate whether there were any differences in efficacy for alleviating UI symptoms between the two types of bariatric surgeries (LSG and OAGB).

## 2. Materials and Methods

This prospective, single-centre study was conducted at the Medical University of Gdańsk in the Department of General, Endocrine, and Transplant Surgery and in the Department of Urology. The bariatric surgery commenced in January 2018 and concluded in December 2018. The follow-up data collection occurred from January 2023 to December 2023. Consequently, two ethical approvals were obtained from an independent ethics committee (the Bioethics Committee for Scientific Research at the Medical University of Gdansk: the first on 8 December 2017 (NKBBN/521/2017), and the second extending the validity of consent from 9 December 2022 (NKBBN/792/2022)). All participants provided written informed consent. The study enrolled women with obesity presenting with urinary incontinence symptoms who had undergone bariatric surgery.

The inclusion criteria were as follows: age > 18 years, BMI > 30 kg/m^2^ with no permanent weight loss achieved, and patients qualified for bariatric surgery who accepted the proposed method of treatment. The exclusion criteria were as follows: any type of urinary incontinence treatment (pharmacological or surgical) before or after bariatric surgery and neurological diseases that may cause lower urinary tract disorders.

Upon admission to the hospital, a thorough medical interview was conducted to gather information on comorbidities, current medications, past surgical procedures, pregnancies and births, and addictions such as smoking. Prior to surgery, patients’ body weight, height, and body mass index (BMI) were measured. The choice of surgical procedure, whether laparoscopic sleeve gastrectomy (LSG) or one anastomosis gastric bypass (OAGB), was left to the discretion of the operating surgeon.

Patients completed the International Consultation on Incontinence Questionnaire-Urinary Incontinence Short Form (ICIQ-UI SF) (score range 0–21) [16] and the Urogenital Distress Inventory (UDI-6) (score range 0–100) [17,18], at the time of their admission for the surgery. Both questionnaires are validated for use in Polish. Furthermore, based on the ICIQ-UI SF, the severity of UI was categorized as slight (1–5), moderate (6–12), severe (13–18), or very severe (19–21). Five years later, the patients completed the same questionnaire and recorded their body weight, height, and body mass index (BMI). The cure rate was determined by scores of 0 on the ICIQ-UI SF. Scores of 0 on the UDI-6 were not anticipated due to its inclusion of questions pertaining to symptoms not exclusively related to UI, such as pain or discomfort in the lower abdominal, pelvic, or genital area. Consequently, for the UDI-6, significant reductions were considered to indicate cures.

The primary endpoint was the 5-year efficacy of bariatric surgery in reducing UI symptoms. Secondary endpoints encompassed the influence of comorbidities (diabetes mellitus type II, hypertension, tachycardia, hypercholesterolemia, sleep apnea, menopause), types of deliveries (vaginal and C-sections), and other abdominal or gynecological operations, as well as a comparison of the effectiveness of two methods of bariatric treatment (LSG vs. OAGB) on the reduction of UI symptoms. Exploratory endpoints involved the effectiveness of the surgery depending on the type of UI (SUI, MUI, or UUI), and also depending on the severity of UI based on ICIQ-UI SF results (slight, moderate, severe, or very severe).

### Statistical Methods

Statistical analyses were performed using GraphPad Prism v.6.07 (GraphPad Software, Inc., Boston, MA, USA) and Statistica v. 13 (Tibco Software, Inc., Palo Alto, CA, USA). A sample size of 34 patients was determined to be necessary to detect a post-operative difference of 3 points on the ICIQ-UI SF, with 95% power and a 5% level of significance, assuming a standard deviation on the ICIQ-UI SF equal to 4.

The Gaussian distribution was checked using the D’Agostino–Pearson omnibus normality test. At least one of the variables failed the test, therefore we decided to use non-parametric tests. Intergroup comparisons between the amounts of values in groups were performed using Fisher’s 2 × 2 exact test. Intergroup associations were checked using the non-parametric Spearman’s correlation test. Numerical values were compared using ANOVAs and the Mann–Whitney U-test for unpaired data (i.e., between post-op non-UI and UI patients) or the Wilcoxon test for paired data (i.e., between UI pre- and post-op survey values). To examine the potential influence of demographic factors (age, weight, and BMI), physiological factors (child delivery and menopause), clinical factors (diseases: DMII, hypertension, and sleep apnea), or type of surgery on the occurrence of UI, the Cox–Mantel proportional hazard regression model was employed. First, a univariate Cox regression analysis was performed for each variable. We set the observation time according to surveys and non-UI as 0 and for UI as 1. Variables with a *p*-value < 0.05 were included in the multivariate Cox regression analysis, with variable selection conducted via backward elimination. All associations are presented as hazard ratios (HRs) with 95% confidence intervals (CIs) and *p*-values (**). Statistical significance was set at *p* < 0.05.

## 3. Results

A total of 124 women undergoing bariatric surgery for obesity were diagnosed with urinary incontinence. However, 32 (25.81%) did not respond to follow-up. Ultimately 92 (74.19%) qualified for the project. The patients underwent two types of bariatric surgery. Demographic data from the 92 patients included in the project were collected before the procedure. They are all summarized in Table 1.

### 3.1. Patient Characteristic

**Figure 1:**
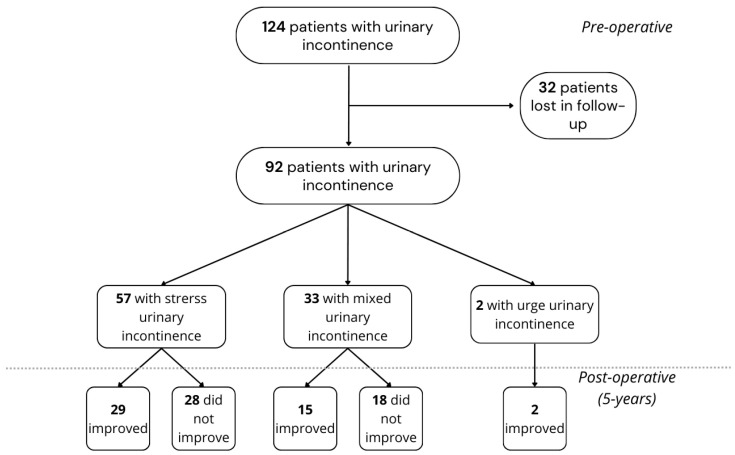
Flow chart of study group classifications and effects of bariatric surgery on urinary incontinence (chart created with Canva).

The study group classifications and the effects of bariatric surgery on UI have been presented as a flow chart (Figure 1, upper panel). A total of 92 patients reported experiencing urinary incontinence (UI). These patients were further classified into the following UI groups: fifty-seven (61.96%) having stress urinary incontinence (SUI), thirty-three (35.87%) with mixed urinary incontinence (MUI), and two (2.17%) with urge urinary incontinence (UUI). Moreover, based on the ICIQ-UI SF, the patients were divided into the following severity sub-groups: nineteen (20.65%) presented with slight symptoms, fifty-eight (63.04%) with moderate, thirteen (14.13%) with severe, and two (2.17%) with very severe UI symptoms.

### 3.2. Subtypes and Severity of Urinary Incontinence

After a 5-year follow-up, there was a significant reduction in UI symptoms in all patients (Figure 1, lower panel; *p* < 0.001, 2 × 2 Fisher’s exact test). Cure rates varied among subtypes of UI, with improvements of 50.88% (*n* = 29), 45.45% (*n* = 15), and 100% (*n* = 2) for SUI, MUI, and UUI, respectively. (Figure 1, lower panel). The ICIQ-UI SF and UDI-6 scores of all patients significantly decreased (*p* = 0.01 in both). The data of patients before and after surgery divided by UI occurrence are presented in Table 2. In accordance with ICIQ-UI SF severity levels, eleven (23.91%) patients showed slight, twenty-eight (60.86%) moderate, eleven (23.91%) severe, and one (2.17%) very severe symptoms of UI. Despite the decreased number of UI patients (92 vs. 46), the statistical distribution of the severity of UI symptoms remained unchanged. Following the categorization of UI into SUI, UUI, and MUI in relation to severity levels based on the ICIQ-UI SF and subsequent analysis of UI incidence before and after a 5-year follow-up period, our findings demonstrated a statistically significant improvement in slight and moderate symptoms exclusively within the MUI cohort (zero and twenty-two vs. four and eight patients with slight and moderate symptoms, respectively; *p* = 0.011, 2 × 2 Fisher’s exact test).

Upon comparing individual answers from the UDI-6 and ICIQ-UI SF questionnaires before and after surgery in the group of patients who experienced UI before surgery, it became clear that those related to SUI displayed a statistically significant reduction, which was particularly evident on the UDI-6 (*p* = 0.004). Additionally, on the ICIQ-UI SF questionnaire, there was a tendency toward a reduction in the symptoms characteristic of SUI.

Nevertheless, there were no statistically significant differences in weight or BMI between patients with persistent UI symptoms and those who improved (Table 2). Based on these findings, we conclude that the hypothesis positing that bariatric interventions reduce incontinence symptoms is supported. This outcome is independent of other demographic and clinical variables.

### 3.3. Types of Bariatric Surgery

**Figure 2:**
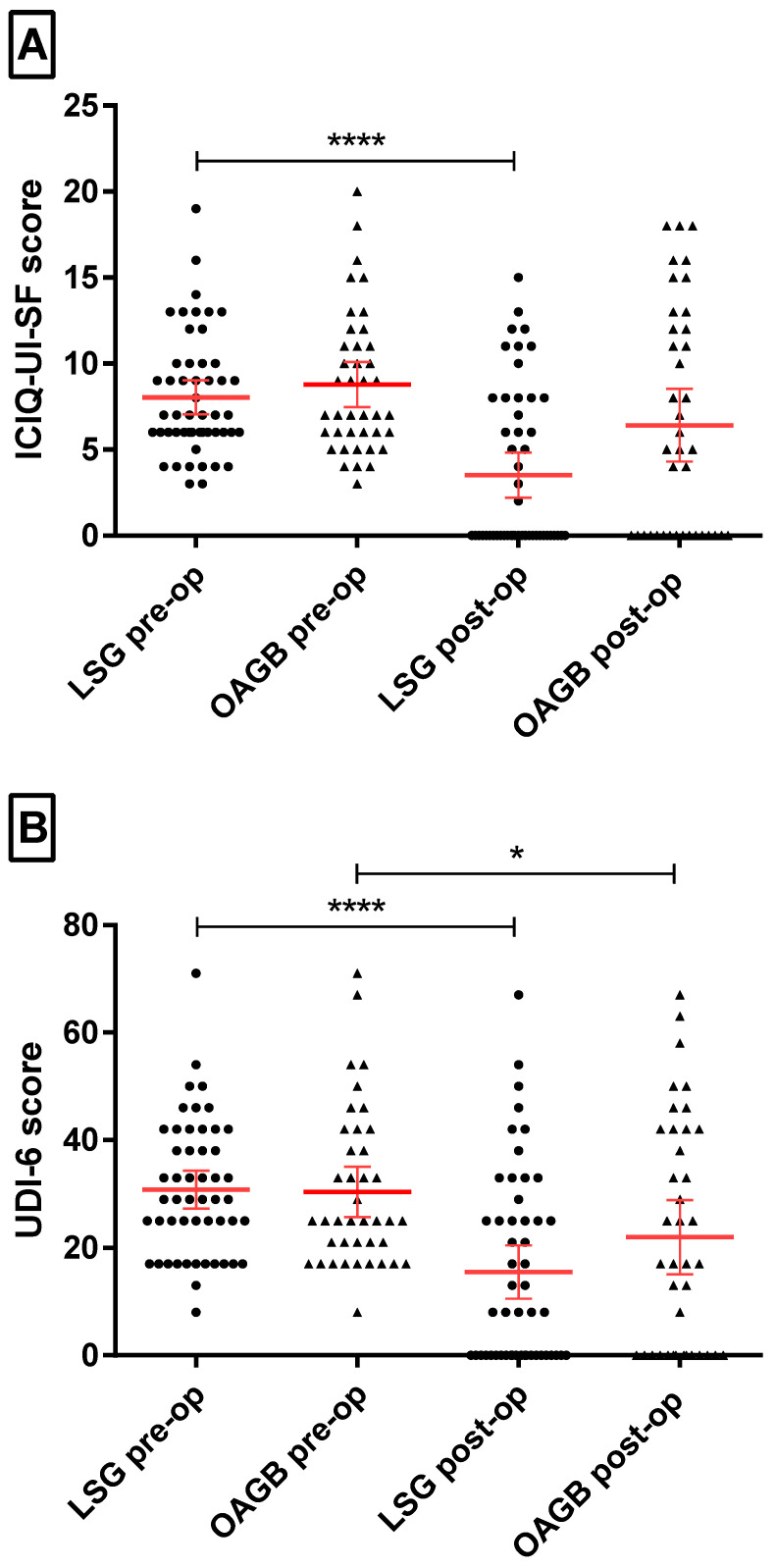
Comparison of incontinence symptom scores in relation to the bariatric procedure used. ICIQ-UI SF (**A**) and UDI-6 (**B**) questionnaire scores were compared for LSG and OAGB surgery before (pre-op) and 5 years after surgery (post-op). Raw data are plotted along with mean values (long horizontal red line) and whiskers—95% of CI. Non-parametric Wilcoxon statistical tests for clustered data (e.g., LSG pre-op vs. LSG post-op) or nonparametric Mann–Whitney U tests for unrelated data (e.g., between LSG and OAGB). *—*p* = 0.05; ****—*p* < 0.001.

Dividing the patients into groups depending on the type of bariatric surgery showed that 52 (56.52%) underwent LSG and 40 (43.48%) underwent OAGB. The study’s findings demonstrated a substantial disparity in efficacy between the two techniques, wherein the LSG technique exhibited a more potent capacity to ameliorate UI symptoms, yet the decrease in BMI in both types of surgery showed no statistically significant difference. UI symptoms were reduced in 29 (55.76%) women after LSG surgery (*p* < 0.001) and in 16 (40%) women after the OAGB procedure (*p* = 0.017). Moreover, in the SUI group after LSG surgery, out of 33 patients, 19 (57.58%) experienced an improvement in symptoms (*p* = 0.002, 2 × 2 Fisher’s exact test) whereas after OAGB surgery improvement showed in only 11 (45.83%) out of 24 patients (*p* = 0.17, 2 × 2 Fisher’s exact test). As mentioned previously, patients with MUI did not experience a statistically significant improvement in the occurrence of UI symptoms. Additionally, a notable difference was observed between the types of surgery in the analysis of the ICIQ-UI SF questionnaire results; there was a twofold decrease in the mean score in patients after LSG (*p* < 0.001), whereas in patients who underwent OAGB, the reduction in score was not statistically significant (*p* = 0.066) (Figure 2A). In contrast, the average UDI-6 score declined significantly regardless of the type of surgery (both *p* < 0.001, Figure 2B), from twofold after 5 years post LSG to a four times lower mean score after the OAGB procedure.

### 3.4. Comorbidities, Surgeries and Births

We analyzed whether other factors may affect the occurrence of UI in patients, regardless of bariatric surgery. Therefore, we assessed comorbidities, including hypertension, diabetes mellitus type 2 (DM2), tachycardia, hypercholesterolemia, obstructive sleep apnea, and menopause as well as previous abdominal and/or gynecological surgery and births, using 2 × 2 Fisher’s exact tests.

Among ninety-two patients, thirty-eight (41.30%) had hypertension, twenty-six (28.26%) had DM2, three (3.26%) had hypercholesterolemia, three (3.26%) had sleep apnea, three (3.26%) had tachycardia, fifty-seven (61.96%) had given birth (vaginal deliveries—*n* = 26, 28.26% and c-sections—*n* = 31, 33.70%), thirty-five (38.04%) had the menopause, and forty (43.48%) had undergone other abdominal and/or gynecological surgery.

In the subgroup of patients who still experienced any form of UI after surgery (*n* = 46; 50%), 39 (84.78%) had at least one of the comorbidities mentioned above. In this group, there were slight differences in the percentages of occurrence of some of the discussed risk factors compared with the previous groups: eighteen (39.13%) had hypertension, twelve (26.09%) had DM2, two (4.35%) had sleep apnea, three (6.52%) had tachycardia, two (4.35%) had hypercholesterolemia, thirty-six (78.26%) had given birth, twenty (43.48%) had the menopause, and twenty-three (50%) had undergone other abdominal and/or gynecological surgical procedures.

The presence of risk factors likely to be associated with the development of UI was assessed using the Cox test. Only two variables from all those previously mentioned showed significant risks of UI: type of bariatric surgery and vaginal delivery. Using univariable tests, we observed that vaginal delivery as well as OAGB surgery presented a significantly higher risk of UI development (HR = 2.26; 1.04–4.92, *p* = 0.03 and HR = 2.155; 1.97–4.12, *p* = 0.04). Furthermore, when a multivariable test was applied, the co-occurrence of vaginal delivery and OAGB surgery presented a 2–3.5 times higher risk (HR = 2.27–3.47, *p* < 0.05) of UI development. Other risk factors did not show any significant HR results. Thus, we accomplished our secondary objective: to examine potential factor(s) influencing the occurrence of UI symptoms in the analyzed groups.

## 4. Discussion

A cohort of 92 patients had urinary incontinence (UI) prior to bariatric surgery. The patients were classified into various UI subtypes: fifty-seven with stress UI (SUI), thirty-three with mixed UI (MUI), and two with urgency UI (UUI). Throughout the 5-year postoperative period, 50% of the women with UI reported complete resolution of symptoms. Additionally, in each UI subtype, we observed the following outcomes: twenty-nine (50%) SUI patients, fifteen (46%) MUI patients, and two (100%) UUI patients experienced a reduction in UI symptoms. Following the classification of UI severity based on the ICIQ-UI SF, only in slight and moderate MUI symptoms showed statistically significant improvement. The current literature lacks sufficient information regarding the long-term effects of bariatric surgery on urinary incontinence. Waeckel et al. conducted a study comparing the effectiveness of bariatric surgery in terms of UI at 1- and 6-year follow-up in a group of 67 women with obesity. A noticeable improvement in the symptoms of stress urinary incontinence (SUI) was evident at one year and this improvement persisted for six years. However, despite the improvement in the symptoms of UUI after one year, there was a deterioration in these symptoms at six years [9]. Anglim et al. enrolled 336 women with obesity, of whom 151 (44%) experienced UI prior to bariatric surgery. Following a one-year observation period, the ICIQ-UI SF and assessment of pad use were conducted on 61 (40%) patients who preoperatively exhibited UI symptoms. The results demonstrated a 40% improvement in UI symptoms. Upon categorizing UI into subtypes, the cure rates were 41%, 38%, and 48% for SUI, UUI, and MUI, respectively [10]. Similarly, Arumugaswamy et al. conducted a study involving 39 patients, with a follow-up period of 1 year. The study reported a 96.5% resolution rate for SUI, which was primarily attributed to decreases in intra-abdominal pressure [11]. Additionally, Bulbuller et al. investigated the effect of LSG on UI in a cohort of 120 women with obesity with a 6-month follow-up period. The overall cure rate for all types of UI was 38%. However, when UI was categorized into subtypes, the cure rates were 61%, 39%, and 25% for SUI, UUI, and MUI, respectively. The efficacy of bariatric surgery was linked to the combined effect of reduced urethral hypermobility and decreased bladder pressure resulting from lower intra-abdominal pressure, which led to more substantial improvements in SUI compared to UUI [12]. According to Swenson et al., it was found that women with obesity with SUI exhibited higher intravesical pressure, thereby necessitating greater demand for the continence mechanism [19]. Additionally, Sugerman et al. have demonstrated a statistically significant decrease in bladder pressure following bariatric surgery [20]. On the other hand, the etiologies of mixed and urge urinary incontinence are more complex, which may account for their lower responsiveness to weight loss [21]. In contrast to prior findings, Nosrati et al.’s study, which involved a cohort of 40 women evaluated before and one year after bariatric surgery, indicated that there were no significant improvements in UI symptoms. However, the study had several limitations, including the assessment of UI based solely on the ICIQ-SF questionnaire, without differentiation among subtypes of UI [15].

Notably, our study highlighted that a decrease in body mass index (BMI) following bariatric surgery was not statistically correlated with a reduction in urinary incontinence symptoms, which was consistent with most previous studies [9,10,14]. On the contrary, some studies have found weak correlations between BMI reduction and UI symptoms after bariatric surgery [11,13]. This incompatibility may be attributed to the fact that BMI is an imperfect indicator of body composition and weight, as it does not account for the distribution of adipose tissue, muscle mass, hydration status, or sex differences. Pang et al. investigated the visceral adipose index (VAI), a more comprehensive indicator for predicting obesity-related comorbidities, and its association with stress urinary incontinence, demonstrating its utility as a tool for assessing the risk of SUI in women with obesity. The VAI incorporates sex, BMI, waist circumference, triglycerides, and high-density lipoprotein cholesterol (HDL) in its calculation, which could be utilized in future studies to assess more precisely the effect of bariatric surgery on UI [22].

Our results suggest that bariatric surgery does not significantly ameliorate severe symptoms of MUI. Statistically significant improvements were observed only in slight and moderate MUI symptoms (as determined by ICIQ-UI SF severity levels). However, SUI and UUI, which are components of MUI, showed substantial reductions in UI symptoms of 50% and 100%, respectively. According to Bunn et al., there exists a connection between the prevalence of overactive bladders (OABs) and lower urinary tract symptoms (LUTS) or metabolic syndrome (MetS) [23]. On the contrary, Zacche et al., through their prospective cohort study involving 840 women, discovered that only obesity acted as a predictor for OAB. Conversely, other metabolic irregularities, such as hypertension, diabetes, and dyslipidemia, exhibited no discernible impacts [24]. However, bariatric surgery, which has the ability to both reduce body weight and induce remission of metabolic syndrome, is anticipated to exhibit a positive impact on all subtypes of UI [25]. Thus, the outcomes of our study can be ascribed to the constrained sample size of 33 patients or the more complex origin of MUI. Future studies should concentrate on enhancing the size of the sample and ensuring equal representation across all subgroups of UI to provide more precise outcomes. Moreover, it is crucial to conduct blood pressure and blood tests for glucose and lipid levels to avoid overlooking any instances of metabolic syndrome.

The effects of comorbidities and previous abdominal or gynecological surgery on the prevalence of urinary incontinence (UI) after bariatric surgery are significant findings. Our results indicate that comorbidities such as diabetes mellitus type II (DM II), hypertension, tachycardia, hypercholesterolemia, menopausal status, and sleep apnea, as well as abdominal and gynecological surgery, do not influence UI symptoms. Bulbuller et al. reported similar findings regarding the influence of DM II and smoking [12]. The same observation was made by Arumugaswamy et al., who examined the impact of DM II, hypertension, and hypothyroidism on UI after bariatric surgery [11]. Conversely, Rodrigues et al. demonstrated the influence of coexistent hypertension, tachycardia, hypercholesterolemia, previous surgery, and obstructive sleep apnea on the persistence of urinary incontinence after bariatric surgery [14]. Furthermore, a recent study by Zhu et al. on approximately 5000 patients reported a significant synergistic effect of hyperlipidemia and obesity on stress urinary incontinence [26].

Our study revealed that among the 57 patients who gave birth, there was a higher risk of persistent UI after bariatric surgery. Similarly, Rodrigues et al. hypothesized the impact of vaginal deliveries and cesarean sections on UI symptoms following bariatric surgery [14]. The occurrence of deliveries in a patient’s history is a well-established risk factor for developing UI, with structural and neuromuscular mechanisms being affected by vaginal delivery [27]. Furthermore, a large cohort study by Tsui WL et al. demonstrated that vaginal deliveries (VDs) pose a higher risk of developing stress urinary incontinence (SUI) compared to cesarean sections (C/Ss) [28]. In light of these findings, the authors recommend early pelvic floor physiotherapy, particularly after VD. These insights are essential for designing effective treatment plans for UI in women with obesity.

Furthermore, our investigation was the first to elucidate a significant difference in efficacy between two modalities of bariatric surgery (LSG and OAGB). LSG surgery exhibited a superior ability to improve UI after a 5-year follow-up period. This phenomenon may be attributed to a set of the most prevalent side effects of OAGB, which are not frequently observed after LSG: gastrointestinal symptoms, including flatulence, diarrhea, and dyspepsia [29]. These symptoms are directly associated with a higher risk of UI [30]. Moreover, patients who underwent OAGB surgery were older than those qualified for LSG (38.9 +/− 10.62 vs. 45.9 +/− 7.67 y/o), which may be associated with the occurrence of menopause and estrogen deficiency. According to Huang et al., who compared 158 perimenopausal and 115 postmenopausal women in relation to SUI, climacteric symptoms are associated with the development of SUI. This association may be attributed to elevated susceptibility to the withdrawal of gonadal steroids or imbalances in central and peripheral neurotransmitters during the menopausal transition [31]. Similarly, Rodrigues et al. considered menopause as the most significant predictive factor for SUI persistence after bariatric surgery [14]. However, our study does not confirm the association of menopausal status with the occurrence of urinary incontinence before bariatric surgery or with persistent UI after bariatric surgery during 5-year follow-up.

In conclusion, this study addresses the knowledge gap concerning the long-term impact (5-year follow-up) of bariatric surgery on UI, particularly in the Central European population, which had not been previously investigated. Bariatric surgery, especially LSG, demonstrates a significant long-term impact on UI symptoms. The effect was more pronounced in SUI than in MUI, likely due to decreased intravesical pressure following bariatric surgery [19,20]. However, MUI cure rates were lower due to its more complex nature. Definitive conclusions regarding UUI cannot be drawn due to the limited sample size of two patients. Clinicians should be aware of the complexity associated with MUI and strive to diagnose and treat overactive bladder symptoms in their early stages. Notably, this study highlighted an issue involving thirty-three MUI and two UUI patients who remained without pharmacological detrusor sedation. The primary clinical implication is that weight loss should be considered the first-line treatment for UI in women with obesity, especially SUI. More invasive treatments, such as placement of mid-urethral slings, regardless of approach (transobturator vs. retropubic), should be considered as a subsequent step if weight loss through diet or bariatric surgery is insufficient to control UI symptoms. Furthermore, it should be noted that evidence of the effectiveness of incontinence surgery in women with obesity is inconclusive [10,32,33].

The main limitation of this study was the absence of urodynamic examinations. Nevertheless, according to EAU Guidelines, there is a strong recommendation against routinely performing urodynamic examinations during the diagnostic and therapeutic process in patients with uncomplicated stress, urge, or mixed urinary incontinence [34]. Consequently, our findings were not significantly affected by this limitation. Recent studies draw attention to not only the use of urodynamic but also additional parameters such as thermodynamic measurements assessing the relationship between pressure and volume obtained during cystometry of micturition [35,36] and viscoelastic measurement, which evaluates compliance of the bladder muscle characterizing the bladder’s storage function [37]. Those parameters might give us a wider understanding of mechanisms and changes taking place in urinary bladder functioning after weight loss associated with bariatric surgery. Furthermore, the absence of pad tests, pharmacological detrusor sedation, and bladder diaries constitute additional limitations. The study lacks a non-surgical control group of women with obesity with UI who did not undergo bariatric surgery. Including such a control group could help ascertain the specific impact of bariatric surgery on UI symptoms compared to natural disease progression or the impact of non-surgical weight loss interventions. Additionally, alcohol consumption, coffee intake, and smoking are factors that can change after bariatric surgery and affect the influence on UI, so this subject should be taken into consideration in future studies. The selection of the type of bariatric surgery has been left to the discretion of the operator. However, this approach could be the source of bias. Consequently, future studies should consider randomizing the choice of surgical procedure, conducted by experienced surgeons. Moreover, the absence of assessment during the follow-up period was intentional to prevent any influence on the patients’ ultimate determination of the presence or absence of urinary incontinence symptoms. Future studies with larger sample sizes, more balanced representation of UI subtypes, and well-designed non-surgical control groups of women with obesity could provide more robust conclusions.

## 5. Conclusions

Bariatric surgery provides a beneficial secondary effect on urinary incontinence (UI) in women with obesity who undergo the procedure for obesity. Furthermore, the laparoscopic sleeve gastrectomy (LSG) procedure should be considered the primary choice for women with obesity experiencing UI with a history of vaginal delivery.

## Figures and Tables

**Table 1:** Demographic data of enrolled patients before the surgery.

Demographic Data of Enrolled Patients		
Patients (*n* = 92)	Criteria	Values
Age [yo]	Mean +/− SD;	43 ± 10
Body weight [kg]	Mean +/− SD;	112 ± 19
Body mass index [kg/m^2^]	Mean +/− SD;	41 ± 6
Vaginal birth	*n* woman (%)	26 (28.26%)
C-section birth	*n* woman (%)	31 (33.70%)
Menopause	*n* (%)	35 (38.04%)
Smoking	*n* (%)	15 (16.30%)
Surgery (non CC, non bariatry)	*n* (%)	40 (43.48%)

**Table 2:** Pre- and postoperative data (abbreviations “pre-op” and “post-op”) of the patients at 5-year follow-up divided by UI occurrence.

Data of the Patients Pre-Op and 5-Year Post-Op Divided by UI Occurence					
	Criteria	Patients Pre-Op *n* = 92	Persistence of UI Symptoms Post-Op *n* = 46	Improvement of UI Symptoms Post-Op *n* = 46	*p* Between Non/UI Post-Op
Body weight [kg]	Median (95% of median CI);	109.5 (105–114)	85 (78–91)	78.5 (75–85)	0.42
∆ weight	Mean +/− SD;	29 ± 20			
Body mass index [kg/m^2]^	Median (95% of median CI);	39.89 (38.06–41.81)	30.04 (28.59–32.18)	29.37 (27.24–32)	0.64
∆BMI	Mean +/− SD;	10 ± 9			
UDI-6	Median (95% of median CI);	29 (25–33)	33 (25–42)	0 (0–0)	<0.001
ICIQ-UI SF	Median (95% of median CI);	7 (6–9)	9 (8–11)	0 (0–0)	<0.001

## Data Availability

The original contributions presented in this study are included in the article. Further inquiries can be directed to the corresponding author.
